# Exploring automatic approach-avoidance tendencies: the impact of self-relevant social feedback on behavior

**DOI:** 10.3389/fpsyg.2025.1556034

**Published:** 2025-03-14

**Authors:** Jinhee Kim, Meeseung Lee, Jihwan Chae, Gahyun Lim, Minyoung Kim, Hackjin Kim

**Affiliations:** Laboratory of Social and Decision Neuroscience, School of Psychology, Korea University, Seoul, South Korea

**Keywords:** approach-avoidance tendency, social evaluation, self-relevance, fear of negative evaluation, touchscreen

## Abstract

Previous studies have reported automatic approach-avoidance tendencies toward various stimuli, such as words, facial expressions, and images in the appetitive or aversive valence domain. This work investigates whether self-relevant evaluative feedback affects these behavioral tendencies using a touchscreen-based approach and avoidance task, in which participants responded to two-colored fish icons either by pulling toward or by pushing away from themselves. Evaluative feedback on participants' personality traits, provided by the fish, served as a task-irrelevant feature. A pronounced valence-congruence effect for positive feedback relative to negative feedback was observed. Interestingly, higher social desirability ratings of social feedback were associated with faster reaction times for approach trials and slower reaction times for avoidance trials. Personality traits were linked to approach tendencies: higher fear of negative evaluation scores predicted a slower approach for both positive and negative feedback compared to neutral feedback. This study demonstrates automatic approach and avoidance tendencies toward self-relevant social feedback, indicating a behavioral predisposition that may be automatically triggered by such feedback. Additionally, this study lays the groundwork for developing touchscreen-based approach-avoidance tasks for measuring individual differences in sensitivity to social feedback and the strength of behavioral predispositions.

## 1 Introduction

In daily interactive environments, people often face various forms of feedback about their performance or character, which can significantly influence many aspects of life, including cognition, social behavior, and mental health. Feedback relevant to oneself is intrinsically salient (Sui et al., 2012) and influences attention allocation (Bargh, 1982). The processing of self-relevant feedback has unique characteristics, making it especially impactful, as it directly affects an individual's self-concept (Swann et al., 1990; McConnell et al., 2009) and emotional responses (Shepperd et al., 1996; Schmitz and Johnson, 2007).

In the emotional domain, conscious or unconscious evaluation of stimuli, such as categorizing experiences in the positive/negative valence domain, is thought to trigger emotions linked to fundamental predispositions to approach positive stimuli or avoid negative stimuli (Fazio et al., 1986; Frijda, 1986; Bradley et al., 2001). These tendencies for appetitive/aversive behaviors in relation to positive/negative stimuli are adaptable for survival in daily life. Aligning with this notion, Lang et al. (1997) proposed two motivational systems where positive stimuli elicit approach behavior, while negative stimuli induce avoidance tendencies. The approach and avoidance task (AAT) is widely used to measure implicit behavioral tendencies. Seminal work by Solarz (1960) using implicit AAT first demonstrated that the valence of words impacts approach/avoidance behavior. This finding has been replicated across countless studies using stimuli such as attitude objects (Chen and Bargh, 1999), adjectives (Wentura et al., 2000; Seibt et al., 2008), and abstract nouns (Citron et al., 2016; Klackl et al., 2023).

Previous studies investigating behavioral tendencies toward social information through AAT have predominantly used facial expressions in both healthy (Rotteveel and Phaf, 2004; Stins et al., 2011; Vrijsen et al., 2013) and clinical populations with depression (Derntl et al., 2011; Radke et al., 2014) and social anxiety (Heuer et al., 2007; Struijs et al., 2017). For example, healthy individuals tended to quickly pull happy faces to themselves and swiftly push angry faces away (Rotteveel and Phaf, 2004; Heuer et al., 2007) when categorizing emotional expressions. Comparing verbal and facial feedback has shown that verbal information is more powerful than facial expressions (Houle-Johnson et al., 2019). Based on this concept, verbal feedback may have a greater impact on approach/avoidance tendencies compared to facial expressions. One study found that when participants judged the valence of adjectives describing personality traits, they demonstrated quicker pulling responses to positive words and faster pushing reactions for negative words (Seibt et al., 2008). Since the context of social interaction is inherently tied to the provider of feedback, it is crucial to study whether approach/avoidance tendencies are elicited by the source of social feedback. No studies have yet assessed approach-avoidance tendencies toward self-relevant verbal feedback presented in the third-person, similar to real-world social evaluations.

The aim of the current study was to develop an implicit manual AAT using a touchscreen interface where participants swiped up (push) or swipe down (pull) a fish icon that provided self-relevant social feedback about their personality traits. Implementing an AAT on a touchscreen monitor takes advantage of the widespread accessibility and flexibility of touchscreen devices such as smartphones and tablets. Previous studies have demonstrated the reliability of touchscreen-based AAT in measuring approach/avoidance tendencies through arm and hand movement (Meule et al., 2019; Rinck et al., 2021; Van Alebeek et al., 2023). The current study had two main objectives. First, we examined whether automatic approach/avoidance tendencies are evident in response to self-relevant social feedback using touchscreen-based AAT. Second, we tested its relevance to individual differences such as fear of negative evaluation, anxiety, or depression to validate this method as a tool for assessing individual sensitivity to social evaluations. This study provides insight into the immediate behavioral responses elicited by social evaluations and valuable implications for mental health interventions, particularly for individuals with heightened sensitivity to social feedback.

## 2 Method

### 2.1 Participants

One hundred and one individuals (average age = 24.07 ± 3.88 years; 65 females) were recruited from the local community from three universities and compensated $15 for participating in a 45-min session. The required sample size was calculated using a power analysis (G^*^Power 3; Faul et al., 2007) with a small effect size (*d* = 0.25), an alpha error probability of 0.05, and a desired power of 80%. All participants had normal or corrected-to-normal vision and no history of psychiatric or neurological disorders. The study protocol was approved by the Institutional Review Board of Korea University, Seoul National University, and Sungkyunkwan University. All participants provided written informed consent.

### 2.2 Materials and apparatus

The behavioral task was programmed and presented using Psychopy (v1.9.6) software and presented on a 10-inch touchscreen (1280 × 800 resolution) placed in landscape orientation at a 10° incline to facilitate optimal visibility.

For each trial, participants were randomly shown second-person statements with one of 150 personality trait adjectives (e.g., “you are kind”) drawn from a standard list of personality trait adjectives by Anderson (1968). This personalization aimed to enhance the psychological impact of social evaluations on participants (Sui et al., 2012). Valence scores of the trait adjectives came from a previous study (Sul et al., 2012), where 80 participants rated the social desirability of individuals possessing each trait using a Seven-point Likert scale, ranging from 1 (highly undesirable) to 7 (highly desirable). From a total of 150 adjectives, 60 were categorized as negative with a mean rating of 3 or less (e.g., “you are cynical,” “you are ruthless”), 60 were categorized as positive with a mean rating of 5 or more (e.g., “you are proactive,” “you are optimistic”), and 30 were considered neutral with mean ratings between 3 and 5 (e.g., “you are gentle,” “you are selfless”).

### 2.3 Mobile approach-avoidance item-swiping task

**Figure 1:**
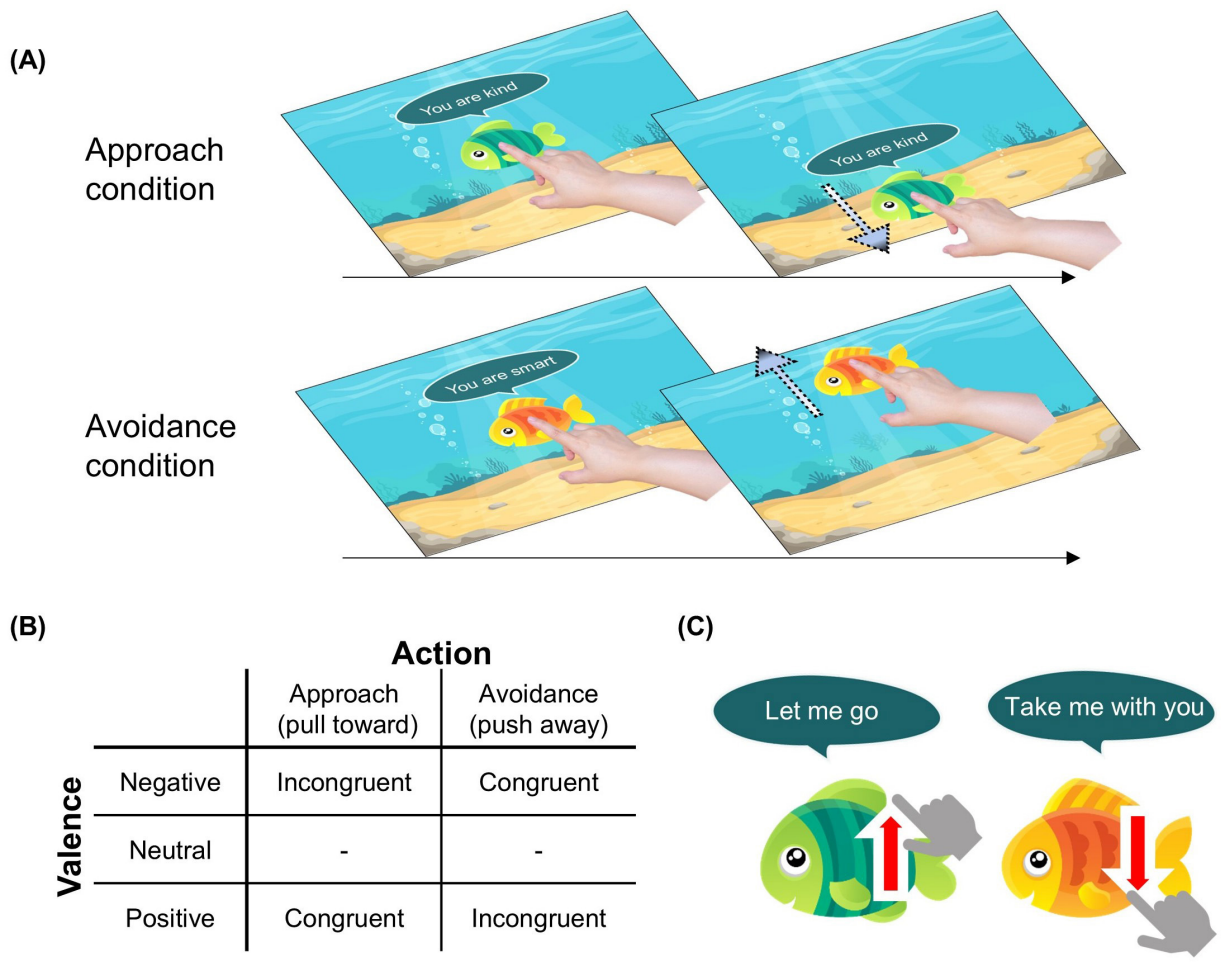
Overview of the experimental paradigm and the social evaluative feedback type. **(A)** During an approach-avoidance item swiping task, a participant was instructed to respond to the color of the fish by pulling/pushing it. **(B)** There were six conditions based on action type × feedback valence and four types based on congruency contingency. **(C)** Filler trials to ensure participants concentrate on the social feedback speech bubble.

This irrelevant-feature AAT measured indirectly automatic bias for social evaluation. It included orange and green fish icons associated with positive, negative, and neutral trait statements in speech bubbles (Figure 1). In this task, participants were instructed:

In this task, you can either pull the fish toward yourself or push it back. Pull the orange fish toward yourself and push the green fish away from yourself. Please ignore any evaluative comments the fish make about you and respond only to the color of the fish. However, if the fish says, “Let me go” or “Take me with you,” follow the request regardless of the fish's color.

The color of the fish to be pulled or pushed was counterbalanced across participants. Upon instruction, participants responded to the color of the fish as it appeared at the center of the touchscreen monitor: green = swipe down (pull response), orange = swipe up (push response). We therefore measured approach as the pull response and avoidance as the push response. Participants used their dominant hands and color-response mapping was counterbalanced. The valence of the social evaluation statements and the associated fish type were presented in a pseudo-random order, with the restriction that no more than three trials with the same valence category (positive/negative/neutral) could occur consecutively.

After 10 practice trials, participants completed 204 trials across five blocks (32, 38, 42, 44, 46 trials) with a short break between blocks. Every trial began with a 1,000 ms fixation, then a fish with a social evaluation statement appeared until response. Feedback (1,000 ms) about correctness followed each response.

To maintain attention on the statements, filler trials prompted push/pull regardless of color, progressively increasing across blocks (Figure 1C; 4, 8, 10, 14, 16 trials). This heightened difficulty and engagement via gamification principles to maintain participant interest and motivation.

### 2.4 Self-report measures and demographic information

After completing the task, participants completed several self-reported questionnaires. Specifically, we administered the Rosenberg Self-Esteem Scale (RSES; Rosenberg, 1965) to assess participant's overall sense of self-worth and the Brief Fear of Negative Evaluation Scale II (BFNE-II; Carleton et al., 2006) to measure a person's tolerance for the possibility he/she may be judged by others. Both constructs—self-esteem and fear of negative evaluation—play a pivotal role in shaping individuals' responses to potential social evaluation, making them critical measures in the context of our approach–avoidance task. We also included the Behavioral Inhibition/Behavioral Activation System (BIS/BAS; Carver and White, 1994) to capture trait-level approach and avoidance tendencies.

Additionally, we assessed depressive and anxious symptoms, given that low mood and heightened anxiety can substantially affect approach–avoidance motivation (Loijen et al., 2020). Specifically, we used the Beck Depression Inventory-II (BDI-II; Park et al., 2020), Center for Epidemiologic Studies Depression (CES-D; Radloff, 1977) scale, and the Patient Health Questionnaire-9 (PHQ-9; Kroenke et al., 2001) to evaluate the severity of depressive symptoms. Anxiety state and trait were measured with Spielberger's State-Trait Anxiety Inventory Form Y (STAI-YS and STAI-YT; Spielberger et al., 1983).

### 2.5 Behavioral data analysis

#### 2.5.1 Data preparation

Dependent variables were response accuracy and reaction time (RT), defined as the latency to move the fish a specific distance after onset. Following prior work (Kersbergen et al., 2015), error trials (i.e., fish pull/push action in the wrong direction), filler trials, RTs < 200 ms or > 2,000 ms, and RTs ± 3 standard deviations (SDs) from the individual mean were excluded. One participant's data was removed due to having >20% trials excluded, and another's data was removed for having >20% filler trial errors, indicating a lack of attention to the social evaluative feedback provided.

#### 2.5.2 RT analysis

RT analyses used R (version 4.2.3) and Python. While participants were instructed to respond based on the fish color, differences between valence types of feedback were measured indirectly.

First, the congruency effect (CE) was calculated separately for positive and negative evaluations (Positive = [approach-positive] – [avoidance-positive]; Negative = [avoidance-negative] – [approach-negative]) (Saraiva et al., 2013; Klackl et al., 2023). Second, mean RTs were calculated for each action (pull vs. push) × valence (positive, neutral, negative) combination. A repeated-measures analysis of variance (ANOVA) was conducted to examine the effects of action and valence. For RT analysis, the multivariate test (Wilks' Λ) was used, as it is preferred over the univariate approach when the assumption of sphericity is violated (O'Brien and Kaiser, 1985). Data are expressed as mean ± SD, and statistical significance was set at *p* < 0.05. For significant main effects and interactions, effect sizes were reported using Cohen's d for pairwise comparisons and partial eta-square (η^2^) for overall effect.

Additionally, linear mixed models investigated the influence of adjective social desirability ratings on RTs for each action (i.e., pull or push), including random intercepts for participants and rounds. To investigate whether more socially desirable evaluations were approached faster or avoided slower than less socially desirable one, separate analyses were conducted for approach and avoidance trials.

#### 2.5.3 Relationships between self-reports and RT differences

RT differences were calculated by contrasting RTs for positive and negative feedback against neutral feedback RTs as the reference. These behavioral scores were then correlated with self-report measures to explore how motivation and personality traits affect approach-avoidance tendencies to social evaluative feedback. Four RT difference scores were computed: Approach toward positive evaluation (App2Pos) = [approach-positive] – [approach-neutral], Approach toward negative evaluation (App2Neg) = [approach-negative] – [approach-neutral], Avoidance toward positive evaluation (Avd2Pos) = [avoidance-positive] – [avoidance-neutral], and Avoidance toward negative evaluation (Avd2Neg) = [avoidance-negative] – [avoidance-neutral]. Partial correlation analyses examined how self-report scores were related to these RT difference scores and CE biases, controlling for gender and age. The present study used a broad range of personality measures in an exploratory fashion. Consequently, separate FDR corrections (*p* < 0.05) were performed for each scale.

## 3 Results

### 3.1 Behavioral results

**Figure 2:**
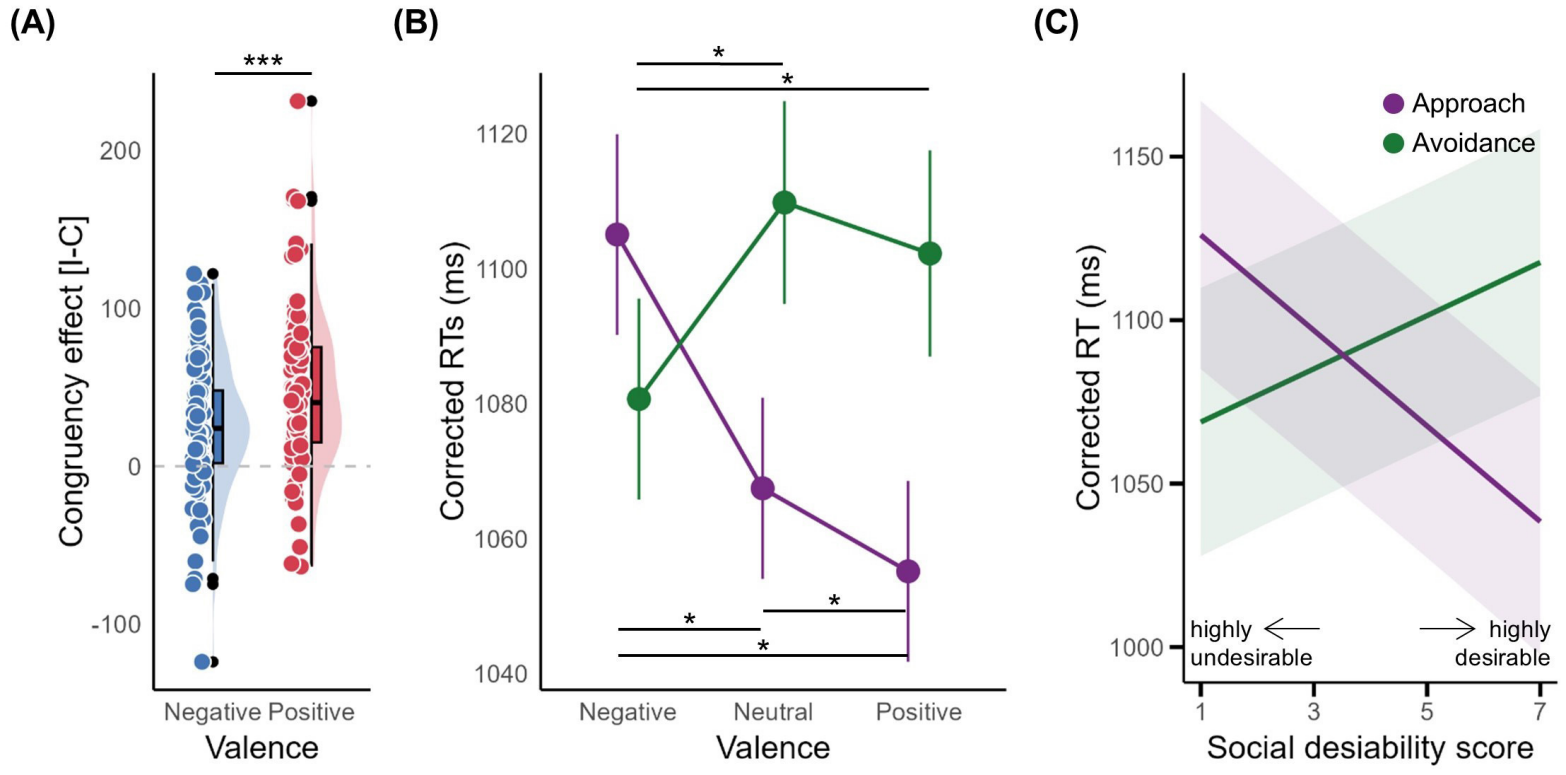
Behavioral results. **(A)** Congruency effect as a function of social feedback valence. **(B)** The mean reaction times (RTs) as a function of action type and social feedback valence. **(C)** Interaction plot demonstrating the effect of action type and adjective social desirability on RTs. The asterisks denote the level of significance: *p < 0.05, **p < 0.01, ***p < 0.001.

The mean error rate was low at 2.82% (SD = 3.53, see Supplementary Table 1), so we focused on RT data. Replicating typical congruency effects (CEs) in the AAT, participants exhibited faster RTs on congruent vs. incongruent trials for positive (M = 47.08, SD = 49.86) and negative feedback (M = 24.40, SD = 42.54; Figure 2A). In particular, the positive feedback CE was significantly larger than the negative feedback CE, *t*_(98)_ = 3.21, *p* = 0.002, *d* = 0.32, 95% CI [0.12, 0.52].

A 2 (Action: approach/avoidance) × 3 (Valence: negative/neutral/positive) MANOVA on RTs showed significant main effects of Action, Wilks' Λ = 0.73, *F*_(1, 98)_ = 36.36, *p* < 0.001, η^2^ = 0.27, and Valence, Wilks' Λ = 0.71, *F*_(2, 97)_ = 19.80, *p* < 0.001, η^2^ = 0.29. As illustrated in Figure 2B, participants reacted faster when pulling compared to pushing the fish and demonstrated quicker responses toward positive evaluations vs. negative evaluations. However, these main effects were superseded by a significant Action × Valence interaction effect, Wilks' Λ = 0.39, *F*_(2, 97)_ = 77.42, *p* < 0.001, η^2^ = 0.62. Simple main analysis showed significant Valence effects for both Approach, Wilks' Λ = 0.40, *F*_(2, 97)_ = 72.88, *p* < 0.001, η^2^ = 0.60, and Avoidance, Wilks' Λ = 0.64, *F*_(2, 97)_ = 26.95, *p* < 0.001, η^2^ = 0.36. For Approach, *post-hoc* analyses reveal that RTs were slowest for negative, then neutral, then positive valences (all Bonferroni adjusted *p* < 0.05). For Avoidance, negative valence RTs were faster than neutral/positive (both Bonferroni adjusted *p* < 0.05).

The LMM revealed a significant Action × Social Desirability interaction on RTs, beta = −22.75, 95% CI [−26.14, −19.36], *p* < 0.001; Std. beta = −0.16, 95% CI [−0.19, −0.14]. To probe the Action × Valence interaction, separate analyses were conducted for approach and avoidance trials. As shown in Figure 2C, for approach trials, pulling down the fish with higher social desirability ratings was associated with faster RTs (beta = −14.65, 95% CI [−17.07, −12.23], *p* < 0.001). Conversely, for avoidance trials, pushing the fish away when shown lower desirability ratings was associated with faster RTs (beta = 7.99, 95% CI [5.58, 10.40], *p* < 0.001).

### 3.2 Relationships between self-reports and behavioral tendencies

**Figure 3:**
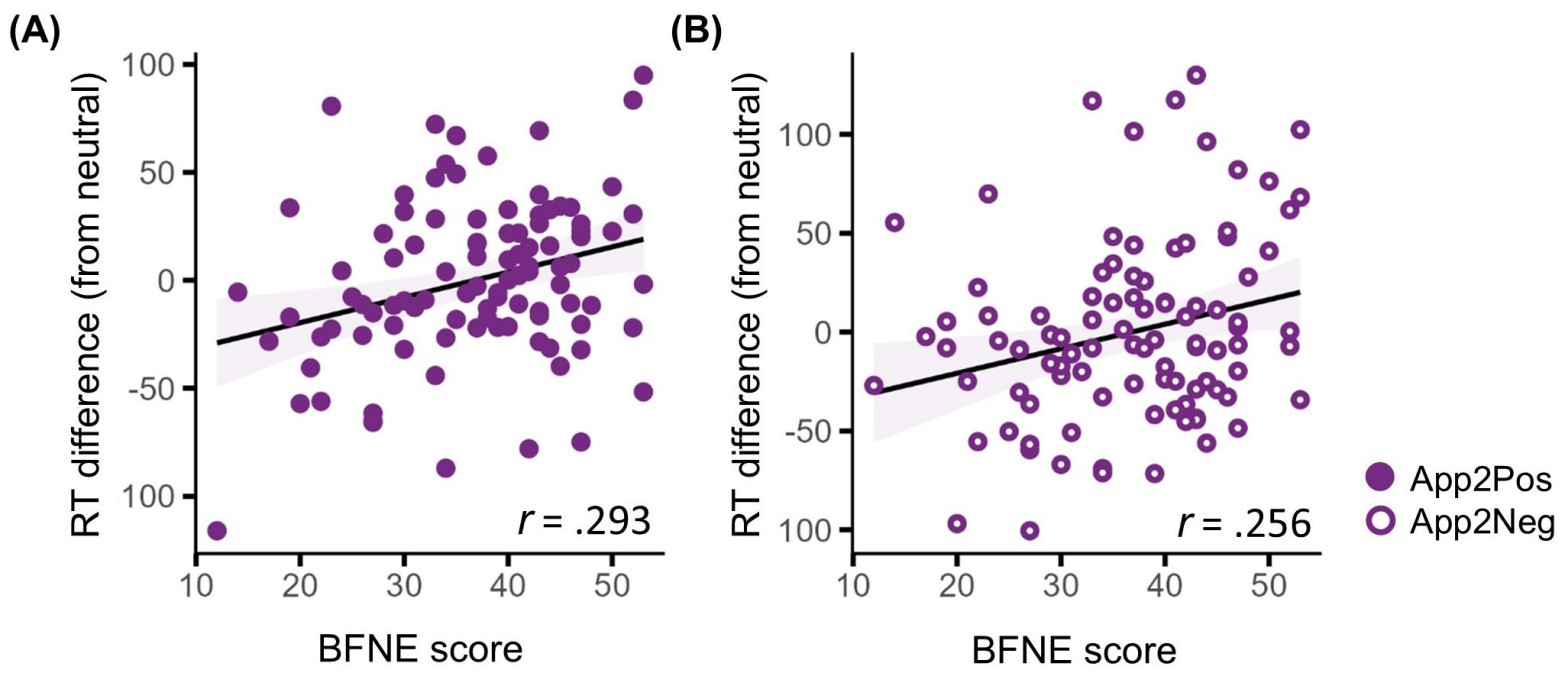
Scatter plots of self-report ratings and AAT behavior indices. **(A)** The correlation between the brief fear of negative evaluation (BFNE) scale and approach toward positive evaluation (App2Pos). **(B)** The correlation between the BFNE scale and approach toward negative evaluation (App2Neg). Filled circle shapes represent positive evaluations, whereas line-filled circle shapes represent negative evaluations. The shaded area around the regression line represents the 95% confidence interval, calculated from the standard error of the fitted values. Values on the y-axis represent marginal means of RTs, adjusted for the effects of age and gender.

Figure 3 shows how personality traits is related to approach-avoidance tendencies for evaluative feedback. Higher scores on the Brief Fear of Negative Evaluation Scale (BFNE) predicted slower approach responses for both positive (App2Pos) and negative feedback (App2Neg) compared to neutral feedback (*r* = 0.293, FDR-corrected *p* = 0.024 for App2Pos and *r* = 0.256, FDR-corrected *p* = 0.036 for App2Neg). BFNE was not significantly correlated with avoidance responses toward negative (Avd2Neg) or positive evaluation (Avd2Pos), *r* = −0.190, FDR-corrected *p* = 0.124 for Avd2Neg, and *r* = −0.123, FDR-corrected *p* = 0.347 for Avd2Pos (Supplementary Figure 1). In addition, no other significant relationships emerged for the remaining self-report measures with any of the other behavioral indices (all FDR-corrected *p* > 0.05).

## 4 Discussion

In the current study, we investigated whether self-relevant feedback affects approach-avoidance tendencies using a touchscreen-based AAT. Participants interacted with an item in the form of a fish that provided social feedback on their character traits by pulling the fish toward themselves (approach) or pushing it away (avoidance) based on color, while their action times and accuracy were evaluated. Consistent with previous studies on the approach and avoidance of written emotional words (Chen and Bargh, 1999; Seibt et al., 2008; Citron et al., 2016), participants quickly approached the fish when receiving positive feedback and quickly avoided the fish when presented with negative feedback. This indicates that self-relevant social feedback independent of the task itself influences approach-avoidance behavior. RT differences were further associated with individual traits and personality measures relevant to evaluation processes in social contexts.

Our study replicated previous findings of valence-congruent behavioral tendencies, demonstrating that participants responded faster on congruent trials (i.e., approaching positive and avoiding negative social evaluations) than on incongruent trials (i.e., approaching negative and avoiding positive social evaluations). This finding aligns with previous studies demonstrating the stimulus-response compatibility phenomenon in where individuals approach appetitive stimuli more rapidly while avoiding aversive stimuli (Solarz, 1960; Chen and Bargh, 1999).

Interestingly, the congruency effect was more pronounced for positive than negative feedback. This finding is consistent with previous research studies reporting stronger stimulus-response compatibility effects with positive stimuli (e.g., positive words, happy faces, appetitive food, and butterflies) compared to negative stimuli (e.g., negative words, angry faces, spoiled food, and spiders) in approach-avoidance tasks (Stins et al., 2011; Klackl et al., 2023). Positive stimuli typically elicit approach behaviors, whereas negative stimuli can trigger various defensive actions, including avoidance (e.g., freezing, rejecting) and aggressive approach behavior (e.g., anger; Lang et al., 1997; Carver and Harmon-Jones, 2009). Additionally, people tend to feel proud after positive self-evaluation but experience mixed emotions during negative self-evaluation (Wang et al., 2022). This could explain why the congruence effect for negative feedback is less evident than for positive feedback.

In the current study, social feedback about the self was utilized as a novel task-irrelevant feature in the context of item-swiping AAT. Previous studies using task-irrelevant AAT found no approach-avoidance bias or weaker biases than task-relevant AAT (Phaf et al., 2014; Kersbergen et al., 2015; Meule et al., 2019). Self-relevant information is known to easily capture attention (Bargh, 1982) and be salient (Sui et al., 2012). Despite being task-irrelevant, the self-relevant social feedback in this study likely drew participants' attention, and resulted in pronounced behavioral tendencies.

Similar to a previous finding that individuals exhibit faster approach reaction times for more likable food (Van Alebeek et al., 2023), we found that reaction times were influenced by the social desirability of the adjectives used as social feedback. Participants quickly approached more socially desirable traits and slowly avoided them compared to less socially desirable traits. This suggests a tendency to pursue socially desirable traits (Edwards, 1953). Our findings provide evidence of such a tendency, known as social desirability bias, demonstrating an automatic approach tendency toward socially desirable personality traits and an avoidance tendency away from socially undesirable ones.

Contrary to our expectations, fear of negative evaluation (FNE) was associated with approach reaction times rather than avoidance. Interestingly, individuals with higher FNE scores showed increased RTs for both positive and negative evaluative conditions compared to the neutral condition. This suggests that individuals with high FNE take longer to pull the fish providing evaluative feedback, possibly due to self-doubt and anxiety in social settings (Watson and Friend, 1969; Van Der Molen et al., 2013). When confronted with emotionally salient evaluations, individuals may engage a self-evaluation process, questioning their worthiness or capability to achieve positive outcomes. This self-doubt can require more processing time, leading to slower approach action times, as observed in the current study. Furthermore, individuals with high FNE are equally sensitive to both self-relevant negative and positive social feedback during social learning (Button et al., 2015), and they also exhibit enhanced elaborative processing of social information compared to those with low FNE, as evidenced by greater parietal P3 event-related potential amplitude in response to both “like” and “dislike” feedback (Zhang et al., 2023). Notably, during approach trials in our paradigm, pulling the fish toward them could be interpreted as actively accepting its evaluation of the participant, a concept that aligns with theories of embodied emotion and cognition. Therefore, beyond mere self-doubt, these findings may also reflect a broader tendency to fear evaluation and cautiously integrate external feedback into one's self-concept in individuals with high FNE scores, thereby contributing to the observed slower reaction times in approach responses.

One limitation of the current study is that the social desirability of the self-referential statements was assessed by an independent group, which may not fully capture individual variations in how participants perceive these statements. Since people can interpret or react to social evaluative statements differently, such individual differences could influence their AAT scores. Future studies could address this issue by obtaining desirability ratings from the same participants who complete the task, allowing researchers to tailor the feedback more accurately to each participant's perceptions of valence and relevance. In addition, given the correlational nature of our findings and the lack of additional physiological or biological measures, it is difficult to draw any causal conclusions regarding the effect of fear of negative evaluation on automatic approach tendencies toward social evaluation. Examining the underlying neural correlates associated with this personality trait (Petrosini et al., 2015) and the pre-behavioral neural circuits using encephalographic recordings of event-related potentials (Sege et al., 2024) could offer deeper insights into how attentional allocation, emotion regulation, and reward sensitivity shape interindividual differences in approach and avoidance tendencies.

This study demonstrated that self-relevant information, such as personality trait evaluation, influences motivational approach-avoidance responses in a touchscreen AAT. Positive social evaluations exert greater motivational power in driving approach behaviors than negative feedback in triggering avoidance tendencies. These tendencies are influenced by the social desirability of personality traits. Our study contributes to understanding how social evaluative feedback influences automatic approach-avoidance behaviors, emphasizing the robust effects of feedback valence on these processes. This study establishes a foundation for the advancement of touchscreen-based AATs, enabling precise measurement of individual differences in sensitivity to social feedback and behavioral predispositions.

## Data Availability

The raw data supporting the conclusions of this article will be made available by the authors, without undue reservation.
