# Supplementary material for: Exploring automatic approach-avoidance tendencies: the impact of self-relevant social feedback on behavior
Source: Front Psychol. 2025 Mar 14;16:1556034. doi: 10.3389/fpsyg.2025.1556034 (PMC11949996; doi:10.3389/fpsyg.2025.1556034)
Supplement: Supplementary file 1 [file Data_Sheet_1.docx]

Supplementary Material

# Supplementary Data

We performed a 2 Action type (approach or avoidance) × 3 Valence (negative, neutral, or positive) MANOVA on accuracy data (Supplementary Table 1). Results showed significant main effects of action [Wilks’ Λ = .67, *F*(1, 98) = 48.27, *p* < .001, η^2^ = .330], feedback valence type [Wilks’ Λ = .92, *F*(2, 97) = 4.02, *p* = .021, η^2^ = .077], and interaction effects [Λ = .90, *F*(2, 97) = 5.40, *p* = .006, η^2^ = .099]. To further probe this interaction, analysis of simple main effect was carried out for each action condition. Simple effects analysis revealed that there was the significant simple main effect in the avoidance condition, Wilks’ Λ = .90, *F*(2, 97) = 5.36, *p* = .006, η^2^ = .099. Post-hoc comparison tests of the avoidance condition revealed significant differences between the negative and positive trial, indicating that participant pushed the fish that provided negative evaluations (error rates = 96.97 %) more accurately compared with the fish provided positive evaluations (error rates = 95.22 %; corrected *p* = 0.006). The simple main effects were not significant in the approach condition, Wilks’ Λ = .98, *F*(2, 97) = .93, *p* = .40, η^2^ = .019.

# Supplementary Tables and Figures

## Supplementary Tables

# Supplementary Table 1. Descriptive statistics of individuals’ accuracy in each of the conditions.

| Action type | Condition | Mean | SD | Min | Max |
| --- | --- | --- | --- | --- | --- |
| Approach | Neutral | 0.99 | 0.021 | 0.87 | 1 |
|  | Negative | 0.99 | 0.016 | 0.93 | 1 |
|  | Positive | 0.99 | 0.014 | 0.93 | 1 |
|  | Filler | 0.97 | 0.043 | 0.78 | 1 |
| Avoidance | Neutral | 0.95 | 0.072 | 0.600 | 1 |
|  | Negative | 0.97 | 0.042 | 0.77 | 1 |
|  | Positive | 0.95 | 0.064 | 0.67 | 1 |
|  | Filler | 0.96 | 0.052 | 0.74 | 1 |
| SD = standard deviation | | | | | |

## Supplementary Figures


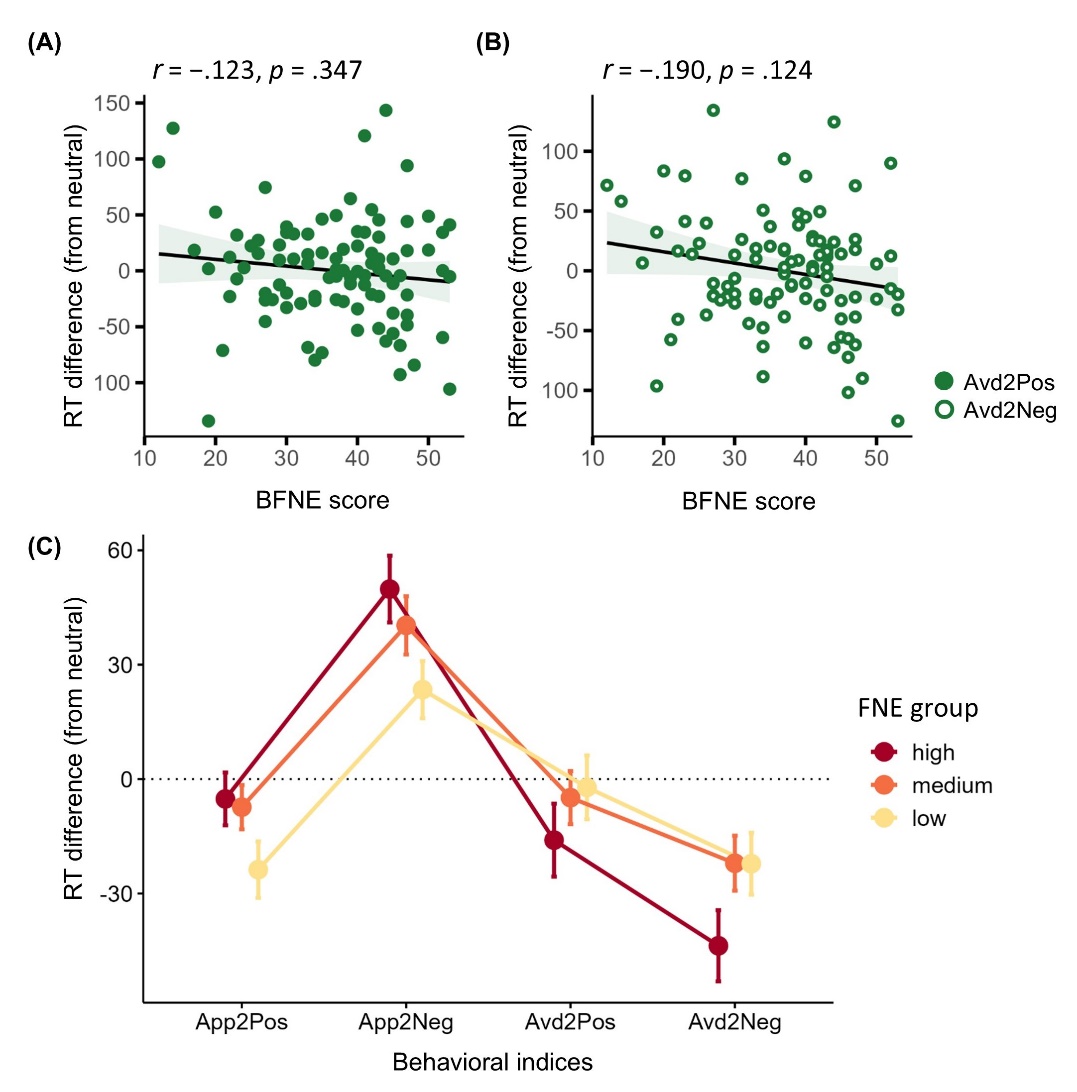


Supplementary Figure 1. The relationship between AAT behavior indices and the brief fear of negative evaluation (BFNE) scale. (A) Scatter plot showing the relationship between BFNE and the avoidance toward positive evaluation (App2Pos) index (*r* = −.123, FDR-corrected *p* = .347). (B) Scatter plot showing the relationship between BFNE and the avoidance toward negative evaluation (App2Neg) index (*r* = −.190, FDR-corrected *p* = .124). (C) Mean reaction time (RT) differences across four behavioral indices (App2Pos, App2Neg, Avd2Pos, Avd2Neg) for each FNE group for visualization purposes. The BFNE score was divided into three groups—low, medium, and high—based on the 33rd and 66th percentiles. A marginal group difference was found in App2Neg, *F*(2, 96) = 2.8, *p* = .064, whereas no significant group differences were observed in the other three indices (all *p* > .10). For (A) and (B), filled circles represent positive evaluations, while line-filled circles represent negative evaluations. The shaded area around the regression line indicates the 95% confidence interval, calculated from the standard error of the fitted values. For (C), data are presented as mean ± standard error. In all panels, values on the y-axis represent marginal means of RTs, adjusted for age and gender.
